# Return on capital? Determinants of counter-migration among early career Israeli STEM researchers

**DOI:** 10.1371/journal.pone.0220609

**Published:** 2019-08-08

**Authors:** Emil Israel, Nir Cohen, Daniel Czamanski

**Affiliations:** 1 Faculty of Architecture and Town Planning, Technion, Technion–Israel Institute of Technology, Haifa, Israel; 2 Samuel Neaman Institute for National Policy Research, Technion, Haifa, Israel; 3 Department of Geography and Environment, Bar Ilan University, Ramat Gan, Israel; 4 Faculty of Economics & Business Administration, Ruppin Academic Center, Emek Hefer, Israel; Universidad Veracruzana, MEXICO

## Abstract

Migration studies emphasize the role of economic, social and cultural capital in shaping out-migration decisions. Yet, little attention is paid to the effect of capital endowment on return migration, particularly among the highly educated. This article examines the extent to which different forms of capital determine return decisions of early-career researchers (ECRs). We hypothesized that individuals from more privileged backgrounds would repatriate at higher rates, due to the benefits that their capital stock might offer them upon homeland re-integration at home. Drawing on a sample of 223 early career Israeli scholars in STEM (Science, Technology, Engineering and Mathematics) disciplines, we used logistic regressions to analyze the effects of material wealth, social ties, and family-oriented cultural capital on their return propensities. No significant differences were found between repatriating and non-repatriating scholars with respect to cultural capital. However, accumulating social and economic capital was positively correlated with the decision to repatriate as was marrying into academic families.

## Introduction

The exponential growth in academic mobility is explained often by the globalization of scientific knowledge, the accelerated development of higher education systems, primarily in the “Global South”, the growing value of international experience in the academic labor market and by an increasingly lenient skilled migration policy, driven by a fierce global race for talent [1–3]. Czaika & Toma [4] recently called for a unified conceptual framework that bridges the long-existing gap in research concerning outmigration and return migration of students and other academics.

Despite a mushrooming literature on the internationalization of mobility among all levels of the academically engaged, from undergraduate to PhD students, and from postgraduates to faculty members [2, 4, 5], research on mobility of early-career researchers–defined as those within 10 years of completing their PhD—has been meager. Save a few notable and primarily policy-driven exceptions about postdocs [6] and other academics who are in the beginning of their research-oriented career [7, 8], determinants, decision-making and trajectories of cross-border mobility among this community of scholars remains largely understudied. Few attempts have been made to examine determinants of return migration among ECRs who have completed their training. While this is congruent with broader tendencies ‘to deal separately, theoretically and empirically, with the issues of migration…and return’ ([2], p. 115) as well as a more specific oversight of academic repatriation (see [9], p. 772), given that the majority of early career scholars, notably international post-docs conceive of their time abroad as ‘risky but unavoidable phase to improve their situation in their home country’ ([6], p. 69), studying their return is long overdue. As Balaž & Williams ([10], p. 218) concluded over a decade ago, ‘the most important significant gap in our knowledge of ISM (international skilled migration) is probably in respect of the process of return’.

Particularly imminent in this respect is the extent to which social networks, and other capital forms, cultural and economic, impact upon return decisions of ECRs. Following Bourdieu [11], economic capital refers primarily to material wealth, while cultural capital is concerned with the assets whose mobilization allows individual social mobility in a stratified environment. Finally, social capital consists of the relationships that persons develop to maintain or advance their standing/position within the current social order. Accumulated at different life-stages, various forms of capital reflect a person’s social class [11,12]. Their accumulation at a person's adolescence and maturation represents his or her social class of origin [13].

The aim of this study is to explore the effects of class of origin on the location decision made by ECRs. Accordingly, it presents an empirical analysis of the determinants of return migration among ECRs. Using the case of Israeli researchers in STEM (Science, Technology, Engineering and Mathematics) disciplines who completed their training abroad, we explore the role played by social, cultural and economic capital in their return decisions. We expect scholars’ social class of origin to affect their repatriation decision. We hypothesize that possessing higher levels of homeland-based social, cultural and economic capital in Israel would lead to higher return propensity, due to the benefits they might offer them upon re-integration in Israel. Thus, and in line with recent calls by migration scholars to take advantage of the ‘availability of survey data (e.g., from online questionnaires) and of secondary datasets to perform statistical analyses’ ([14], p. 135), we examine the ways in which capital affects academic return-migration.

Scholarly research has long acknowledged the salience of social capital in international migration in general [15] and skilled migration more specifically [16]. Studies have shown that international movers are better embedded into social and professional networks at different geographic scales, notably transnational, and typically possess greater levels of social and cultural capital compared with non-movers [17]. However, despite recent evidence that networks, and other non-pecuniary factors (e.g., emotional attachment to the homeland), shape skilled migrants’ decision to return [18, 19], majority of studies remain focused on microeconomic incentives, chiefly income differences between countries.

The study contributes to the literature on international mobility of academics in three distinct ways; first, we push the boundaries of academic mobility, engaging with a professional segment that has received little attention within population geography and related fields. We concur with Ackers ([2], p. 108) who argued that although ‘undergraduate flows may be numerically dominant’, mobility of early career researchers, notably doctoral and post-doctoral researchers ‘may be of greater concern’ due to the investment made in them by their home state as well as their—present and prospective–impact on multiple scientific disciplines. Second, by focusing on return decision of young academics we attempt to do away with the persistent dichotomy between migration and return, which migration scholars have long critiqued [10]. Indeed, following Wang’s [9] claim that ‘very few studies have focused on the return migration of academics’, we conceive return as integral to what Castles [20] termed ‘the migration process’. Finally, by utilizing online questionnaires, our paper sets to expand the breadth of research methods used in the field of academic mobility.

The paper includes five sections. The next section presents review of the literature on academic mobility and the potential role that various forms of capital (i.e. economic, cultural and social) accumulated prior to a scholar's location-choice decision, have on repatriation. The third section present the research hypotheses and the empirical method. The fourth section presents results of our statistical analysis. Section five presents our conclusions and some discussion.

## Academic mobility

Recent years have witnessed growing interest in the migration of academics [21]. Conceived within a broader *mobility turn* in the geography of education and learning [22, 23], studies have examined the mobile nature of individuals engaged in the knowledge-production process. Early research on academic mobility distinguished between students and more experienced researchers [10]. The mobility of the former was understood as a unique case of ‘brain training’, whereas the latter was seen as engaged primarily in the production, or distribution of knowledge. More recent accounts, however, tend to (re)-conceptualize them co-jointly. Thus, King and Raghuram [14], for example, argue that academics conducting research abroad may well be included in the continuum of mobile studentship, ‘because fieldwork, visits to archives, sabbaticals, secondments…all comprise mobility experiences’, and Madge, Raghuram and Noxolo [5] call to do away with binary divisions between the mobility of students and scholars, noting that they are intertwined and ‘implicate us all in international study’ (p. 695). In a recent study, Czaika, and Toma [4] bridge these distinct literatures, suggesting that overseas academic careers are often the outcome of having an international student education.

Conceptual differences aside, research about *knowledge migrants* has highlighted the distinct nature of their mobility [2]. In contrast to most skilled professionals who are *moved* (e.g., through intra-corporate transfers) or follow traditional patterns of supply and demand in the international job market, academic mobility is often self-initiated, driven by the quest for credibility and prestige and ‘stimulated by a desire for *professional socialization’* ([24], p. 26). Mobility is therefore ‘a normal part of scientific life’ ([25], p. 151), and a necessary condition for the formation and maintenance of cross-border social networks that are essential for professional progress and success. Increasingly, mobility of academics is encouraged–and even incentivized–by their home (and host) institutions, as well as national governments who see the merit (and potential economic value) in transnational scientific interactions and exchanges [26, 9]. Indeed, many universities designate international travel funds for graduate students and postdoctoral researchers. Amounts and technical arrangement to access funds (e.g., conference presentation is a pre-requisite in some institutions) change considerably between universities, and even faculties/departments in the same university, but they typically allow young scholars to travel to at least one international conference each year.

Studies have shown that alongside ‘the drive for scientific curiosity’ ([27], p. 18), academic migration is also motivated by other, more economically practical reasons, like wage differentials and contractual insecurity back home [28]. But, as Ackers ([2], p. 106) argues, migrating academics seldom see these in narrow economic terms, but rather views them ‘within the context of wider costs of living (including travelling), social benefits (especially healthcare and childcare), and access to pensions.’ Other motivations may include improving spousal chances of landing lucrative (non/academic) jobs or exposing their children to different cultural milieus [10]. At the macro-scale, some academics quote prevalent corruption or cumbersome bureaucracies in their countries of origin as a reason to out-migrate [29, 30]. Others may be troubled by the changing political climate in their countries of birth, including rising national(istic) sentiments. Cohen and Kranz [31], for example, conclude that some departing Israeli scientists are concerned with the country’s political and religious radicalization and their German counterparts ‘often feel as national misfits’ (p. 806). Other structural and circumstantial factors, including immigration policy in destination countries, macroeconomic conditions or increasing racism also stimulate academic outmigration [32].

Migratory decisions of academics are linked to–and impacted by–their own identities and biographies. Alongside key socio-demographic factors like age, gender, ethnicity and personal status, mobility of scientists is strongly correlated with their life-course and broader career trajectories [33]. Hence, for example, greater and more frequent mobility is expected of early-stage (and younger) academics for the purpose of securing ties with–and establishing themselves as part of–the global scientific community [21]. Studies have shown that female academics tend to be less mobile than their male counter-parts due to social norms that dictate their greater involvement in routine child-rearing activities [34]. Similarly, non-tenured faculty, who typically enjoy limited access to travel-related institutional funding, are at a disadvantage compared with tenured staff [35].

In contrast to the voluminous literature on (out)-migration, much less is known about return-migration of academics. With some recent exceptions [9] questions pertaining to their determinants of return and the extent to which they might differ from those who choose to stay abroad, have not been adequately explored. Conceptually, studies typically drew on neoclassical economics and the new economics of labor migration (NELM) to explain return of academics. The former, seeing out-migration as a long-term strategy for personal and professional gains, conceives of return as a failure to attain one’s goals. While non-pecuniary considerations (e.g., racial persecution or home sickness) may sometimes explain the decision to return–and thereby forego success in destination countries, neoclassical economists normally prioritize monetary factors, which help increase accumulation of human capital and dis-incentivizes return [36]. Within the framework of NELM, outmigration is a household strategy aimed at diversifying and minimizing risks associated with dysfunctional markets in the country of origin. Repatriation is therefore a normative response, which under certain conditions could benefit both the returning individual (if his or her accumulated experience overseas could be put to better use back home) as well as household members left behind. However, if circumstances change (e.g., old parents pass away), return may be delayed or prevented altogether [37].

The dominance of economistic theorizing in (return) migration research has recently been challenged [38, 39]. In response, scholars have sought a more balanced analysis of microeconomic and social factors that drive the return of skilled migrants in general and academics in particular. Some studies highlighted the salience of transnational social relations in international students’ post-graduation decision-making process [40] and the effect of scientific linkages with the home country on the probability of return among researchers working abroad [41]. Others examined similarly the role of tertiary education institutions in the return migration process [9] as well as that of family considerations and, to a lesser extent, government policies in both sending and receiving countries [18]. Overall, this body of research shows that academic mobilities may take distinct forms whose motivations may be quite unique in comparison with other types of skilled migration [21]. However, much like other skilled professionals, academics are not immune from travel-related hardships (e.g., integration into scientific ‘culture’ in the host society) and their mobilities are always ‘embedded in employment relations and social and cultural contexts’ ([21], p. 83).

To explore the embeddedness of academic migrants and decipher the ways in which mobility becomes a form of capital in the academic labor market, scholars have often employed Bourdieu’s [11,12, 42] notions of social and cultural capital [43,44]. Bourdieu has explored class reproduction, accentuating combinations of capital (economic, cultural and social) that create a 'social space', a subtle expression of class stratification [12]. Whereas economic capital refers to material wealth, cultural capital is concerned with educational qualifications, familiarity with the languages and the arts, as well as more general "know-how" that is often attributed the social context of his or her upbringing (e.g. parents’ occupations and social class of origin, place of residence. See: [45]). Finally, social capital refers to the ties and connections individuals garner through membership in (more-or-less structured) social networks and which can be used to maintain or advance their affiliation with a given social class [46].

All three forms of capital play a significant role in ‘status attainment’ [47]. Their relative amounts determine one’s position in the prevailing social structure. Thus, higher levels of capital translate into greater chances of social ‘success.’ Bourdieu notes, however, that no one form can fully explain the individual’s social order. Rather, it takes all three forms. In that manner, social success includes the wealth of a person’s cultural capital, such as academic achievement [48], and favorable employment outcomes, that leads to better opportunities to accumulate economic capital [49]. The recognition and valuation of one’s cultural capital is directly enabled by his or her social capital, and the latter often facilitates the accumulation of the former.

The structure and volume of capital that a person accumulates must be contextualized within his or her ‘social trajectory’ ([50], p. 99). That is, the effectiveness of using capital for different social benefits is contingent upon the context and people’s abilities to apply it most effectively. The context, for Bourdieu, relates to the ways in which social space is divided into different fields of action, such as economic, housing, education, and academic fields [51]. Accumulating capital forms within these fields forges a habitus [50], a ‘feel for the game’ [52], or an intimate familiarity with the social field. The habitus introduces a valuable acquaintance regarding, for example, desirable behaviors as well as needed actions that a person needs to preserve a social position, or to advance in the social order that the field dictates [11]. Academia as a social field has “its own logic” ([53], p. 53), and it ‘recognizes those who recognize it’ ([53], p. 101). That is, young scholars who wish to develop an academic career need ‘to be willing and able to play by the rules of the game if they want to be included as members of the institution’ ([54], p. 63), and to acquire professional skills and knowledge about the nature of academic work that ‘are essential for a successful entrance into an academic career’ ([54], p. 61). In relation to the aim of the present study, this prerequisite raises the question of social class of origin and how it effects ECRs odds.

Studies have shown that social background affects individual educational attainments, implying that parental (notably cultural) capital is a reliable predictor of academic success [13]. Accordingly, young students, who are familiar with rules of the academic field (e.g., colleges and universities), may devise strategies that would assist them in acting–and succeeding—within it [55]. Studying abroad, for example, is a valuable expression of having a ‘feel for the game’ in this specific field [56, 57], ‘an element of the academic habitus’ ([21], p. 87), that advances mobility in the field. Cultural capital was found to be raising the likelihood studying abroad as well as the decision to specialize in prestigious disciplines and institutions [58]. Similarly, meager economic resources were in negative correlation with the likelihood of studying overseas [33].

A small body of knowledge currently exists that examines the role of social class among academics in decisions to repatriate. In a recent article, Labrianidis and Vogiatzis [58], for instance, found a higher propensity of repatriation among Greek scholars abroad who hailed from families with greater levels of economic capital. Material wealth had a facilitative role because it enabled them to endure longer periods of unemployment upon return to the homeland. In other cases, cultural capital earned overseas was found to be important in decisions to return migrate. Waters [23] showed that studying in prestigious North-American universities equipped Chinese students with cultural capital, whose exchange value upon return meant landing better-paying positions at esteemed local institutions. Similarly, stronger and denser home-based social networks were positively correlated with return-migration, whereas weak(er) social ties produced a reversed effect [59, 60].

National cultural identity, such as family and friendship values, increased for example young scholars’ willingness to repatriate and to rejoin those values [61]. Cultural comfort in the sending country facilitates repatriation as it provides reassurance and familiarity with language and social environment [62]. In other cases, such as those that are associated with Chinese students, repatriation is associated with their willingness to care for aging parents [61, 63] or because of their parents’ opinion [64]. These findings resonate with Granovetter’s [65] notion of embeddedness, that is the extent to which integration into personal networks may prove beneficial for job seekers who could gain valuable information about employment opportunities (e.g., be tipped off before specific job openings). Acquiring such information depended on social ligatures, or ‘weak ties’ [66], namely extensive but weaker social connections maintained with distant acquaintances like friends of friends or fellow alumni [67]. This way of applying social capital is common among ‘social elites’, where ties are first developed, for example, while attending esteemed institutions of higher education. They are later sustained through elaborated alumni networks that affect employment experiences and, consequently, opportunities for social mobility [68, 69].

These findings notwithstanding, there is still relatively little research about the links between ECRs' class of origins and their career development. That is, between their social, cultural and economic forms of capital, possessed by academics abroad and their trajectories of repatriation. Specifically, little is known about the role played by these forms of capital in decisions made by early career researchers. Given the importance of mobility for the professional advancement of scientists, primarily those in the early stages of their career in STEM disciplines, and in light of the fierce competition over tenured positions in both home and host countries, examining the variegated factors which accelerate or otherwise inhibit scientific (return) migration is imperative.

## The empirical method

### The hypotheses

The social stratification analysis proposed by Bourdieu provides the theoretical basis for understanding repatriation choices made by ECRs. We hypothesize that various forms of capital possessed by ERCs affect their decisions.

#### Hypothesis 1

A positive relationship exists between a scholar’s economic capital, measured as material wealth, and the probability to repatriate. Failing to return to the home country may jeopardize a person’s heretofore accumulated wealth, particularly housing. This is especially true when the country of origin is less developed than the destination country and wealth accumulation requires great effort. However, the specific macroeconomic conditions in the origin and host countries at the time that the decision is to be taken, may affect the decision.

#### Hypothesis 2

A positive relationship exists between a scholar's social capital in the sending country to repatriation. The scholar’s social capital is comprised of academic, communal and familial ties. Networking in the academy enables him or her to forge weak ties in this social field [66] and could positively affect the decision to repatriate. Better positioning in the community alleviates repatriates’ post-graduation difficulties [61]. Finally, close involvement with friends and family members (e.g. parents, siblings) is expected to positively affect the decision to repatriate.

#### Hypothesis 3

A positive relationship exists between the position of the scholar's parents in the social space and the decision to repatriate. Utilizing cultural capital in the most effective way, requires intimately familiarity with the field in which the form of capital is applied [50]. In this regard, familiarity with the academy could be essential for an individual who plans an academic career. Parents' position in the social space can positively affect this familiarity. A scholar who was brought up in–and is familiar with—an academic environment is likelier to repatriate.

### Model specification

We test the proposed hypotheses by means of the well-known logit model in which the dependent variable is dichotomous and indicates the decision to repatriate (1) or not (0). It is indicated by Z_*nij*_ where scholar *n* who studied in country *i* and is living in country *j* chooses to repatriate. Nine explanatory variables are hypothesized to influence the choice probability in Eq (1):

$$\text{Z}nij=\beta_{0}+\overset{2}{\underset{o=1}{\sum }}\;\beta_{o}AS_{no}+\;\overset{4}{\underset{x=1}{\sum }}\;\beta_{x+2}ECN_{nx}+\overset{5}{\underset{j=1}{\sum }}\beta_{j+6}HLP_{nj}+\overset{2}{\underset{y=1}{\sum }}\beta_{y+11}ASS_{ny}+\overset{5}{\underset{l=0}{\sum }}\beta_{l+13}PRS_{nl}+\overset{5}{\underset{d=1}{\sum }}\;\beta_{d+18}S_{nd}+\overset{5}{\underset{d=1}{\sum }}\;\beta_{z+23}CN_{nv}+\overset{2}{\underset{w=1}{\sum }}\beta_{w+28}PRT_{nw}+\overset{5}{\underset{w=1}{\sum }}\beta_{w+30}L_{cn}+\varepsilon_{nij}$$ (1)

*β*_*1*_, …, *β*_*n*_ are parameters to be estimated and *ε*_nij_ is an error term.

*AS*_*no*_ is a binary variable denoting possession of economic capital in the form of a house by scholar *n* at the time of the decision to repatriate or remain abroad. 1 indicates yes and 0 otherwise.

*ECN*_*nx*_ is a set of dummy effect variables denoting a subjective assessment of the scholar’s economic status at level *x* at time of the decision. The level of economic status is indicated by *x*_*1*_ = not good; *x*_*2*_ = below average; *x*_*3*_ = average; *x*_*4*_ = good or very good (reference group).

*HLP*_*ni*_ is a set of dummy effect variables denoting the level j of guidance and advice (social capital), which friends and parents provided as for whether to repatriate. The level of social capital is indicated by *j*_*1*_ = did not assisted; *j*_*2*_ = slightly assisted; *j*_*3*_ = assisted to some extent; *j*_*4*_ = assisted to a great extent; *j*_*5*_ = assisted to a very great extent (reference group).

*ASS*_*ny*_ is a binary variable denoting a scholar’s answer *y* as to whether the hiring institution assisted in spousal job search (social capital), where 1 indicates yes; and 0 otherwise (did not assist).

*PRS*_*nl*_ is a set of dummy social capital effect variables denoting the level *l* of routine assistance given to a scholar *n* by his or her parents (and by his or her spouse's parents, if relevant) such as financial aid, childcare, etc. (l = 1, …, 5), where *l*_*1*_ = do not assist at all; *l*_*2*_ = assist slightly; *l*_*3*_ = assist to some extent; *l*_*4*_ = assist to a great extent; *l*_*5*_ = assist to a very great extent (reference group).

*S*_*nd*_ is a set of dummy variables that indicate the degree of *d* of the geographic proximity of the scholar’s *n* parents (or of his or her spouse) live (social capital) (d = 1, …, 5); where *d*_*1*_ = Not close; *d*_*2*_ = partial regional closeness, *d*_*3*_ = regional closeness, *d*_*4*_ = close proximity, *d*_*5*_ = full proximity. The degree of closeness indicates whether the parents and/or the spouse’s parents live in the same city and/or region.

*CN*_*nv*_ is a dummy variable indicating the level *v* (v = 1, …, 5) of importance in terms of Granovetter's weak ties, in obtaining the scholar’s *n* current job (social capital); where *c*_*1*_ = not important at all, *c*_*2*_ = not so important, *c*_*3*_ = important to a small extent, *c*_*4*_ = very important, *c*_5_ = extremely important (reference group).

*PRT*_*nw*_ is a binary variable that denotes a scholar’s answer *w* as to whether his or her parents or spouse’s parents are faculty members (cultural capital) 1 indicates yes and 0 otherwise (not faculty).

*L*_*cn*_ is a dummy variable indicating the level *c* (c = 1, …, 5) of importance of an unsatisfactory information concerning job opportunities in the academia, thus affecting the decision of scholar *n* to permanently leave his or her home country, or to repatriate (cultural capital); where *c*_*1*_ = not important at all, *c*_*2*_ = not so important, *c*_*3*_ = important to a small extent, *c*_*4*_ = very important, *c*_5_ = extremely important (reference group).

### The research population and data collection

The hypotheses were tested by reference to Israeli ECRs who specialized mainly in STEM disciplines. A handful of exceptions included researchers in architecture, economics and business trained in STEM-oriented institutions (e.g., The Technion).

Israeli investment in STEM sectors has long been credited as being responsible for the country’s economic prosperity [70] and state investment in the national higher education system shows a clear preference for academic training in these disciplines. It is partly for this reason that Israeli PhD-holders in most STEM disciplines outnumber those in the humanities and social sciences [71]. Only a fraction of STEM PhD holders eventually obtains a tenured position at one of the nation’s eight universities. The postdoc abroad has become a necessary, although insufficient, condition for obtaining a tenure-track position. Not surprisingly, it is often women, ethnic minorities–both Jewish (e.g., Mizrahim- Jews originating from Muslim countries) and non-Jewish (Arabs), as well as graduates from disadvantageous socioeconomic backgrounds for which the postdoc–and other types of early career training—abroad is a particularly daunting challenge [72, 73].

Considering these characteristics, the capital available to young researchers is of crucial importance, both in the decision to leave for (post)doctoral training abroad or postdoctoral studies, as well as to return. Hence, the study’s custom-designed questionnaire, included questions about a scholar’s capital stock (in origin and destination countries) at the time of decision making. The questionnaire also included questions about his or her current position (e.g. faculty, postdoc, industry, etc.), background characteristics (e.g., personal status, having children, home and work address, academic qualifications, postdocs internships, and academic and professional interactions while studying). Other questions focused on the informant’s community life, his or her social relationships with friends and parents as well as parental education and professions.

The study had been approved by the guidelines set forth by the Samuel Neaman Institute for National Policy Research’s (SNI) ethics committee (made up of the institute’s board members and headed by its Chair) and in line with those accepted in the social sciences. The survey, conducted between October 2015 and February 2016, drew on a sample of early career Israeli STEM researchers, who were living either in Israel or abroad at the time and who (notably) became faculty members in the previous few years. The study initially identified recent Israeli repatriating scholars, by utilizing two main data sources. First, was a list of 180 scholars who recently joined the Technion–Israel Institute of Technology, which is home to the largest number of STEM researchers in the country. This inventory specifically concentrated on individuals who joined the Technion during the three years prior to the survey. In addition, the Technion provided a list of few dozen scholars who at the time were candidates and who eventually were not hired. To draw information about young STEM scholars that recently joined other institutions in Israel, we scrutinized these institutions' websites. Combining these three mentioned sources enabled to detect 330 faculty who returned to Israel (repatriates).

We further searched websites of universities and research-oriented institutes in the US, Canada and Europe, in order to identify Israeli scholars who are employed there. Their biographies were then reviewed to assess whether they were, indeed, Israeli. Finally, we used LinkedIn to identify Israeli scholars who are employed as faculty members at universities abroad. Our search resulted in a list of 113 Israeli scholars in STEM disciplines who live abroad (i.e non- repatriates).

Drawing on the revealed behavior of the sampled scholars, two groups of ECRs were constructed. The first consisted of individuals who–when surveyed—had already returned and were living in Israel. A second was made up of individuals who were living abroad when surveyed. The compiled database, which included a total of 443 Israeli ECRs (non/repatriates) with their email addresses, allowed us to attach a link that accessed the questionnaire to the sampled scholars. Ascertaining whether sampled ECRs were successful in obtaining faculty positions was possible only if they had taken the survey and filled out the questionnaire.

## Empirical results

### Sample characteristics

The survey yielded 223 valid questionnaires (a response rate of 51%). 66.4% of respondents (148) decided to repatriate after their (PhD) graduation or at the end of their post-doctoral training. 33.6% (75) of those sampled decided to migrate, that is leave Israel permanently. Most of them currently reside in the USA (66.6% of sampled non-migrants), where most (59.6%) sampled scholars underwent their post-doctoral training.

Table 1 indicates that 47% of sampled scholars, are from the sciences, 35.4% are engineers, and a much smaller group (12.6%) are trained in the social sciences (mainly in architecture and economics). 91.9% of the respondents became faculty members. The results indicate that while repatriates and non-repatriates exhibit similar patterns of professional development (Table 1), the share of faculty among non-repatriates is smaller than repatriates (85.3% and 95.3%, respectively).

**Table 1:** Respondents' social, demographical, economic and professional characteristics.

Variable	Categories (%)						
Gender	**Male**	**Female**	**Total**	**N**			
Non-repatriates	81.3%	18.7%	100.0%	75			
Repatriates	79.7%	20.3%	100.0%	148			
Total	80.3%	19.7%	100.0%	223			
Ethnic origin	**Europe and North America**	Asia and Africa*	**Total**	**N**			
Non-repatriates	82.7%	17.3%	100.0%	75			
Repatriates	83.1%	16.9%	100.0%	148			
Total	83.0%	17.0%	100.0%	223			
Matrimonial status**	**Non- Married**	**Married**	**Total**	**N**			
Non-repatriates	17.3%	82.7%	100.0%	75			
Repatriates	17.8%	82.2%	100.0%	148			
Total	17.6%	82.4%	100.0%	223			
Having children**	**No**	**Yes**	**Total**	**N**			
Non-repatriates	24.0%	76.0%	100.0%	75			
Repatriates	18.2%	81.8%	100.0%	148			
Total	20.2%	79.8%	100.0%	223			
General discipline	**Sciences**	**Engineering**	**Social Sciences**	**Medical Sciences**	**Unknown**	**Total**	**N**
Non-repatriates	42.7%	36.0%	17.3%	2.7%	1.3%	100.0%	75
Repatriates	48.6%	35.1%	10.1%	4.7%	1.4%	100.0%	148
Total	46.6%	35.4%	12.6%	4.0%	1.3%	100.0%	223
Contemporary professional status	**Faculty Member**	**Postdoc**	**Adjunct Faculty Member**	**Industry/** **Privet market**	**Total**	**N**	
Non-repatriates	85.3%	8.0%	4.0%	2.7%	100.0%	75	
Repatriates	95.3%	2.7%	0.0%	2.0%	100.0%	148	
Total	91.9%	4.5%	1.3%	2.2%	100.0%	223	
Indicated economic status**	**Not Good**	**Below Average**	**At the Average**	**Good to Very good**	**Total**	**N**	
Non-repatriates	2.7%	14.7%	40.0%	42.7%	100.0%	75	
Repatriates	0.7%	3.4%	36.5%	59.5%	100.0%	148	
Total	1.3%	7.2%	37.7%	53.8%	100.0%	223	
Duration of post-doctoral training	**No post-doc**	**A year**	**2–3 years**	**4–5 years**	**6+ years**	**Total**	**N**
Non-repatriates	20.0%	10.7%	29.3%	28.0%	12.0%	100.0%	75
Repatriates	6.1%	5.4%	48.6%	27.0%	12.8%	100.0%	148
Total	10.8%	7.2%	42.2%	27.4%	12.6%	100.0%	223

* Including Israeli-Palestinians scholars that were sampled in the study’s survey.

** At time of decision making.

Internships (PhD or postdoctoral studies) lasted 3.23 years on average. It should be noted that groups were not significantly different with respect to time spent abroad, gender, ethnic origin, and familial status at time of decision making (Table 1). 82.4% were married at the time of decision making. 79.8% had children. These figures should be understood within socio-cultural context in Israel, where due to the mandatory three-year military service, ECRs are typically older than colleagues in other countries. As a result, a sizeable share of them are married with children to support [74, 75].

80.3% of sampled scholars were males. In Israel, only 29% of faculty members are women, while in STEM disciplines their share is even lower, including Engineering and Architecture (14%), Physical sciences (13%), and Mathematics and Computer Sciences (11%) [72]. The situation is more extreme for ethnic minorities, with Arabs, who make up one fifth of the country’s population, constituting less than 3% of senior faculty and Mizrahim–less than 10% [72, 73]. Table 1 indicates that most sampled scholars (repatriates and non-repatriates) are Ashkenazim (Jews originating from Europe and America), and as such, compatible with the ethnic composition of Israel’s scientific community. These findings are congruent with the general literature, which shows that many young scholars originate from a privileged social status, which facilitate their engagement in international mobility [58].

### Results

Initially we analyzed the hypothesized relationships by means of Mann-Whitney a-parametric test and *χ*^2^ tests (Tables 2 and 3).*χ*^2^ tests were utilized for independent variables that were measured in a nominal scale (S1 Appendix). The independent variables in the Mann-Whitney tests were scores that the sampled scholars provided in an ordinal scale. The results indicate a significant difference in the capital stock of the two types of the sampled scholars. The most significant differences were observed within the social and economic forms of capital. S1 Appendix provides a supplemental data that summarizes the main variables used in the analysis of the research data and in the construction of the statistical models.

**Table 2:** Group differences between non-repatriating and repatriating scholars (Mann-Whitney tests).

Variable	Group Belonging	Sample Proportion	Mean	Mann-Whitney U-test
Geo closeness of the parents (PRTS_PRX)	Non-repatriates	75	83.30	Z = -5.165
	Repatriates	148	126.54	Sig. = 0.000
Lack of adequate information concerning job opportunities in the non-selected country (LKINFO)	Non-repatriates	75	132.32	Z = -3.789
	Repatriates	148	101.70	Sig. = 0.000
The importance of friendships and personal contacts in obtaining the current job (CONCT)	Non-repatriates	75	98.77	Z = -9.175
	Repatriates	148	118.71	Sig. = 0.025
The degree to which friends and parents assisted in decision making (ADVC_PRFRD)	Non-repatriates	75	62.94	Z = -4.856
	Repatriates	148	100.75	Sig. = 0.000
The degree that parents (and his or her spouse's parents) routinely assist their offspring (e.g. in funding, child rearing and education) (ASST_PRS)	Non-repatriates	75	72.03	Z = -6.768
	Repatriates	148	132.25	Sig. = 0.000
Specifying the economic status upon decision making (ECO_STUS2)	Non-repatriates	75	96.10	Z = -2.947
	Repatriates	148	120.06	Sig. = 0.003

**Table 3:** Group differences between non-repatriating and repatriating scholars (Chi-Square tests)*.

Variable	Categories (%)			
**Has the institution assisted in spousal job search? (**ASST_INST)**	**No**	**Yes**	**Total**	**N**
Non-repatriates	67.7%	32.3%	100.0%	65
Repatriates	90.4%	9.6%	100.0%	125
Total	82.6%	17.4%	100.0%	190
*Statistical test*	*χ*^2^ = 15.48, df = 1 p≤ 0.000			
**At least one of the scholar's parents is or was a faculty (**PART_ACD**)**	**No**	**Yes**	**Total**	**N**
Non-repatriates	81.3%	18.7%	100.0%	75
Repatriates	79.7%	20.3%	100.0%	148
Total	80.3%	19.7%	100.0%	223
*Statistical test*	*χ*^2^ = 0.08, df = 1 p*χ*^2^ 0.860			
**At least one of the in-laws is or was a professional (**PART_PROF**)**	**No**	**Yes**	**Total**	**N**
Non-repatriates	16.0%	84.0%	100.0%	75
Repatriates	20.3%	79.7%	100.0%	148
Total	18.8%	81.2%	100.0%	223
*Statistical test*	*χ*^2^ = 0.594, df = 1 p≤ 0.441			
**At least one of the in-laws is or was a faculty (**PARTSPS_ACD**)**	**No**	**Yes**	**Total**	**N**
Non-repatriates	94.6%	5.4%	100.0%	56
Repatriates	86.1%	13.9%	100.0%	115
Total	88.9%	11.1%	100.0%	171
*Statistical test*	*χ*^2^ = 2.8, df = 1 p≤ 0.095			
**At least one of the spouse's parents is or was a professional (**PARTSPS_PROF**)**	**No**	**Yes**	**Total**	**N**
Non-repatriates	29.3%	70.7%	100.0%	58
Repatriates	27.9%	72.1%	100.0%	122
Total	28.3%	71.7%	100.0%	180
*Statistical test*	*χ*^2^ = 0.4, df = 1 p≤ 0.487			
**An owner of a dwelling asset, at time of decision (**OWN_HOUS**)**	**No**	**Yes**	**Total**	**N**
Non-repatriates	77.3%	22.7%	100.0%	75
Repatriates	43.2%	56.8%	100.0%	148
Total	54.7%	45.3%	100.0%	223
*Statistical test*	*χ*^2^ = 23.04, df = 1 p≤ 0.000			

* Some of the variables in the table exhibit an incomplete sample (that is, a total lower than 223 respondents), since some of the ECRs were single at time of the survey, without accompanying spouse.

** The institution in which the scholar works at (or was employed right after his or her graduation/postdoc).

Analysis of property and location of home ownership indicate that repatriates benefit from a better positioning in comparison to non-repatriates. For example, the latter have a lower average score in the variable ECO_STUS2. This indicates that repatriates were significantly wealthier at the time of decision making than non-repatriates (p<0.05, Z value of −2.9). Furthermore, repatriates were more financially invested in Israel. About 57% of them owned a property there at time of decision making in comparison with 22.7% among non-repatriates (that is, were not homeowners, not in the destination country and nor in the origin country).

This variable of home ownership partially explains the wealthier status reported by repatriates, in comparison to non-repatriates. Home ownership is an important feature of young person's wealth [76, 77] indicating—among other things—the economic resources that are available to him or her while abroad as well as upon return. In contemporary Israel, home ownership distinguishes between wealthier and less fortunate young individuals. Although home ownership rates are relatively high (approximately 70% according to Gruber [78]), recent data indicate that (first) home acquisition becomes an immensely difficult task, especially for young families. Sluggish real income and skyrocketing property values have made home ownership a privilege of the wealthier strata in Israel [79, 80]. For most young Israeli families, parental assistance is often prerequired in order to purchase their first homes [81]. Hence, some of the sampled ECRs may have kept properties in Israel as a capital accumulation strategy which allows them to use the money obtained from renting it. This doesn’t mean that they left Israel with an intention to return-migrate, since keeping the property—and renting it—yields fair monetary return. Alternatively, keeping the property could enable returnees a smoother reintegration process by using the accumulated rent–or the property itself–upon return. Either way, it is clear that these financial strategies are not available for ECRs who own no property in Israel.

Owning an apartment or house, is also an important factor in location decision. A house that stands at a person's disposal, delineates his or her socio-spatial vicinity and the social networks that can be utilized for their purposes. This is especially valuable in the initial stages of the repatriation process since it alleviates the burden of re-integration and assimilation in the receiving society. Similarly, in comparison to non-repatriates, repatriates benefit from an augmented parental support. Obviously, they benefit from improved embeddedness in the community field of their country. The variable of ASST_PRS indicates the intensity of parental assistance after repatriation and the role of family in facilitating the repatriates’ life during this period. Repatriating scholars seem to be benefiting from an emotionally, economically and socially meaningful network, especially in terms of child rearing and financial support (see Table 2).

These results raise the question of cause and effect. Does knowledge about parents’ ability and willingness to assist affect a scholar’s decision, or does this assistance occur subsequently. Parents’ willingness to assist could be a post-hoc reaction to the scholar’s decision. The variable ADVC_PRFRD indicates a possible direction. There is a significant difference (p<0.001) between the two sampled groups (see Table 2). Repatriates were more influenced in their location choices, from guidance and advices provided by their parents and friends (Z value of −4.9).

At the same time, the results indicate that cultural capital is not significantly different between groups. Both repatriates and non-repatriates possess similar shares of parents that are or were faculty members or professionals. These findings are not surprising since individuals in both groups benefit from high degree of cultural capital, defined by their qualifications and scholarly achievements. The literature indicates that most of them reproduce their parents' social class [55, 58]. Both groups have parents that worked or are still working in professional/White collar occupations. Also, their spouses’ parents seem to be of the same status (Table 3). However, there is a significant difference (p<0.10) between repatriates and non-repatriates in the variable of PARTSPS_ACD. The share of spouses whose parent(s) are (or were) faculty members is greater among repatriates (14%) than non-repatriates (5.5%). Additionally, it should be noted that personal considerations that may affected the scholars’ location choices and regard circumstances in which parents are involved were also examined (e.g., sick family members, old parents). However, due to their marginal importance for most of the respondents, they were not included in the analysis.

The results indicate that scholar’s network that specifically assists in obtaining valuable knowledge on faculty opportunities are significantly different between the two-sampled groups. For example, the variable LKINFO indicates that non-repatriates obtained a higher average score than repatriates. The significant difference (p<0.001) indicates that those that decided to permanently leave Israel in favor of an alternative career overseas suffered from insufficient knowledge and lacked personal relationships that could help them secure academic jobs in Israel. The variable of CONCT that indicates the importance of early acquaintance with key persons in the local academia, in relation to a successful candidacy for faculty, further emphasizes how those that permanently left Israel, suffered from socio-professional shortage that could assisted to obtain better results. The latter, however, seem to benefit from what can be a possible compensation that partially overcomes their relatively shortage in socio-professional networking in Israel. The variable of ASST_INST in Table 3 indicates that the share of scholars who left Israel and were assisted by the institution in which they were hired abroad in getting a job for their spouses is significantly (p<0.001) larger than returnees (32.3% and 9.6%, respectively).

Table 4 presents the results of two binary logistic models that tested the relation between scholars’ decision to repatriate and the explanatory variables. Controlling for the variables entered, the models allowed an examination of the net effect of a scholar's capital stock on the chances to repatriate.

**Table 4:** Model estimation results (LOGIT): Dependent variable = ECR’s repatriation.

	**Model fit summery**	**Model 1**		**Model 2**	
	Number of observations	223		223	
	–2 Log-likelihood	93.425		125.354	
	Cox & Snell pseudo-R^2^	0.435		0.323	
	Nagelkerke pseudo-R^2^	0.606		0.450	
	**Parameter**	**Estimate**	**SE**	**Estimate**	**SE**
Economic Capital	Owning assets OWN_HOUS)			1.250	0.478**
	Specifying the economic status upon decision making (ECO_STUS2)			0.479	0.274*
Social Capital	The degree to which friends and parents assisted in decision making (ADVC_PRFRD)	1.062	0.301***	0.825	0.227**
	Has the institution assisted in spousal job search? (ASST_INST)	-2.746	0.776***		
	Parental supporting (ASST_PRS)	1.049	0.266***		
	Geo closeness of the parents (PRTS_PRX)	0.283	0.167*	0.435	0.157**
Cultural Capital	Lack of adequate information concerning job opportunities in the non-selected country (LKINFO)	-0.693	0.262**	-0.644	0.208**
	In-laws are from the academy (PARTSPS_ACD)	1.883	0.954*	1.718	0.869*
	Constant	-2.977	0.919**	-2.940	1.130**

Note: *Significant at the 0.10 level;

**significant at the 0.05 level;

***significant at the 0.01 level.

Model 1 is stronger than model 2, since the effect of the independent variables in it are statistically highly significant at the 95% or 99% level, while the effect of some of the independent variables in the second model are statistically significant at a lower level –90%-95%. The results show that the scholar’s decision where to live and work is associated with capital stock. In both models, social capital is more important than other forms of capital.

Model 2 shows that the accumulation of economic capital is positively related to repatriation (Table 4). Both OWN_HOUS and ECO_STUS2 are significant. These variables imply that economic capital could mitigate post-repatriation challenges, like sustained periods of unemployment, both of the ERC and spouse. For example, affluent scholars can utilize their economic ability to remain unemployed for longer periods, especially when there is a spouse involved. The opposite effect is evidenced among scholars who are less financially endowed, thus confirming hypothesis number 1.

Scholars who were influenced more from close social circles in their decision (ADVC_PRFRD) have greater chances to repatriate (with a level of significant p<0.001 in model 1 and p<0.05 in model 2). This finding is supported by the clear, significant and positive association between a decision to repatriate and scholars (and spouses, when relevant) who are routinely assisted by their parents, monetarily or in other forms (e.g., childrearing) (ASST_PRS variable in model 1). Parental support is also related to their geographical proximity, as PRTS_PRX indicates in the estimated models (Table 4). The closer the scholar’s (and his or her spouse’s) parents, the more likely it is for the scholar decision to repatriate (a parameter that is positive and statistically significant in level of p<0.10 in model 1, and p<0.05 in model 2). These results confirm our second hypothesis, and as such corroborate previous studies which showed that strong social ties with friends and family impact upon mobility decisions of academics [33, 82].

It is interesting to note that the probability to return to Israel is significantly declining (p<0.001), when the hiring institution assists in finding a job for his or her spouse (Model 1). Institutional support (ASST_INST) seems to be less important for repatriates than for non-repatriates. This result can be explained by the shortage in social, economic and cultural capital forms (both abroad and in Israel) among non-repatriates, which could otherwise encourage their return. The results support the second hypothesis that indicated how the scholar's inferior positions within the academy and community fields of the sending country can hamper his or her repatriation.

The differences between the examined models, indicate that a substitute relationship exists between the social and economic forms of capital, when relating to the chances of an ECR repatriation. While model 2 contains two economic capital variables (OWN_HOUS and ECO_STUS2) and does not include two variables that represent the social capital (ASST_INST and ASST_PRS), model 1 presents exactly the opposite. Both of these aforementioned social capital variables, affect the scholar’s potential material wealth (the potential earning capacity of the spouse, and the household's expenditures and incomes pattern that is affected through parents' assistance), thus indicating on reducing the economic capital effects on the chances of an ECR's repatriation.

The cultural capital effect on the ECR’s repatriation is manifested by PARTSPS_ACD. It was found to be a relatively weak indicator that affects the probabilities of a scholar to repatriate. Nevertheless, the variable carries a positive effect on the probability for a scholar to repatriate. Benefiting from the existence of a faculty member within the extended family that lives in the sending country enhances the scholar's chances of returning home (p<0.10 in both models). The potential to enjoy from valuable guidance, in regarding to how to manage an academic career, especially in its initial stages, can explain the result that further confirms the study's third hypothesis. It is interesting to note how this connotes with the estimation of LKINFO. On both models (Table 4), the estimation of the variable reveals a negative and significant relationship (p<0.05) with the chances to repatriate. It seems that the chances to stay abroad grow with the lack of satisfactory information concerning job opportunities within the Israeli academia. One can assume that a better mentoring here (e.g. of a family member or past academic supervisors) can add valuable insights for the scholar, as for his or her chances to be faculty, and thus to significantly affecting decision making of the scholar.

## Conclusions

The article explores the role played by class of origin in location decisions of early-career researchers. Drawing on a sample of Israeli researchers in STEM disciplines who had been living abroad, we examined the extent to which the accumulation of capital forms (e.g. socio-professional networks, material wealth and cultural ‘know how’) impact upon decision to repatriate.

No significant differences were found between individuals who repatriated and those who opted to remain abroad with respect to a host of personal and professional factors, including gender, ethnicity, marital status, academic discipline, and duration of academic training abroad (e.g., doctoral studies). In addition, we found no significant differences between the cultural capital of parents, suggesting that to a large extent, members of both groups originate from families with strong educational and professional backgrounds and, therefore, reproduce their parental social class.

These similarities, however, conceal differences with respect to other forms of capital held by group members, including economic and social, which effect their return probabilities. Specifically, repatriates showed significantly higher levels of economic and social capital accumulation in comparison to non-repatriates. For example, when compared to their non-repatriating co-nationals, repatriates were more financially invested in Israel, as manifested by their higher rates of home ownership in the country at the time of return decision-making, Furthermore, repatriates were found to benefit from improved social ties and networks in Israel, as evident by their reported routine parental support and stronger social (e.g., friendships) and professional (e.g., in academic circles) relationships, which helped them obtain their current position. These findings indicate that greater endowments of capital had a facilitative effect on scholars' decision to repatriate.

Our findings suggest that while each form of capital is important in and of itself, to explain repatriation we must consider their simultaneous operation. Therefore, to successfully apply their accumulated cultural capital in the academic field, ECRs need to concurrently have significant levels of capital in other domains, namely economic (e.g., own property) or social (e.g., live in proximity to–and rely upon the help of—family). Capital interdependence among researchers means that insufficient endowments of social and/or economic capital could hamper the successful deployment of their cultural capital within the academic field in Israel, which could lend itself to obtaining a faculty position.

Though our study centers on the Israeli contexts, its findings could be generalized in two important ways; first, in an ever more diversifying academia, ECRs class affiliation becomes increasingly meaningful [83]. It affects the scholar’s trajectories of mobility and location-choices as well as access to information regarding, for example, scholarships and other funding sources. Second, since mobility is by now a pre-requisite for academic career, Israel should not be conceived as *Siu Generis*, regardless of its geopolitics and/or geo-economics. Our findings stress the need to carefully consider the distinct mechanisms of stratification that explain social class differences, and their impact upon scholars’ decisions. Specifically, given that such mechanisms vary across geo-cultural contexts, we expect they would lend themselves to different manifestations of capital forms and, consequently, return decision. Thus, for example, whereas home ownership rate is a salient characteristic of economic capital in Israel, and therefore a critical determinant of ECRs repatriation, this may not be the case in other countries where renting is a more common household strategy. Future studies should take account of these contextual factors as they explore return trajectories of academics.

Our analysis makes use of the revealed behavior approach, which gauges the actual behavior of researchers rather than their real preferences. As such, revealed behavior can express compromises in persons' location choices, ensuing from different considerations and limitations, as for example, accepting what the market has to offer, or other personal constrains, that foster the neglect of alternate selections [84]. Future research may well utilize the stated preferences approach to overcome rational bounded decisions of the sampled population. That is, surveys in which researchers select their preferred choices from a list of hypothetical alternatives. Such surveys would allow them to avoid real-world limitations [85]. Further studies also need to be carried out with a sample from other disciplines, beyond STEM, to examine whether there are any differences in terms of researchers' return patterns and their capital stock that affect their decisions. To conclude, and in acknowledging the potential contribution of this study, we invite further discussions and explorations in the interest of fostering robust debate on the role of class of origin on migration of highly skilled human capital.

## Supporting information

S1 Appendix Variables, measurement scales, descriptive statistics and explanations. (DOCX)

S2 Appendix Bivariate correlation matrix between explanatory variables. (DOCX)
