# Correction: Return on capital? Determinants of counter-migration among early career Israeli STEM researchers

**DOI:** 10.1371/journal.pone.0221882

**Published:** 2019-08-26

**Authors:** Emil Israel, Nir Cohen, Daniel Czamanski

An incorrect version of S1 Appendix was published in error. Please view the correct S1 Appendix below.

## Supporting information

S1 Appendix Variables, measurement scales, descriptive statistics and explanations. (DOCX)
