# Supplementary material for: Return on capital? Determinants of counter-migration among early career Israeli STEM researchers
Source: PLoS One. 2019 Aug 8;14(8):e0220609. doi: 10.1371/journal.pone.0220609 (PMC6687145; doi:10.1371/journal.pone.0220609)
Supplement: S1 Appendix — (DOCX) [file pone.0220609.s001.docx]

**Caption:**

S1 Appendix: Variables, Measurement Scales, Descriptive statistics and Explanations

| **Form of Capital** | **Variable Code** | **The Variable’s Meaning** | **Mean** | **S.D.** | **Scale of Measurement** |
| --- | --- | --- | --- | --- | --- |
| Economic Capital | ECO_STUS2 | Specifying the economic status upon decision making | 3.4 | 0.0 | Ordinal scale of: 1.’Not good’ to 4. ‘Good and even very good’. |
|  | OWN_HOUS | An owner of a dwelling asset, at time of decision if to terminally leaving Israel or to repatriate to it | 0.5 | 0.0 | Dichotomic nominal scale of: 0. 'No'. 1. 'Yes'. |
| Culture Capital | LKINFO | The importance of inadequate information about tenure track opportunities in the non-selected country, in determining if to terminally leaving Israel or to repatriate to it | 1.8 | 0.1 | Likert ordinal scale: 1. ‘Not important at all’ to 5. ‘Very much Important’. |
|  | PART_ACD | At least one of the scholar's parents is or was a faculty | 0.2 | 0.0 | Dichotomic nominal scale of: 0. 'No'. 1. 'Yes'. |
|  | PART_PROF | At least one of the in-laws is or was a professional | 0.8 | 0. 0 | The same as the above variable. |
|  | PARTSPS_ACD | At least one of the in-laws is or was a faculty | 0.1 | 0.0 | The same as the above variable. |
|  | PARTSPS_PROF | At least one of the spouse's parents is or was a professional | 0.7 | 0.0 | The same as the above variable. |
| Social Capital | PRTS_PRX | Degree of geographic proximity to which a scholar’s parents (or the parents of his or her spouse) are living to his or her place of living | 2.3 | 0.1 | Ordinal scale of: 1.’No closeness’. 2. 'Partial closeness (one of the parents' couples live in the region)'. 3. 'Fully partial closeness (two pairs of parents live in the region)'. 4. 'Close proximity (one of the parents' couples live in the locality of the scholar and his or her spouse)'. 5. 'Full proximity (all the parents from all sides live in the locality of the scholar and his or her spouse)'. |
|  | CONCT | The importance of friendships and personal contacts in obtaining the current job | 2.5 | 0.1 | Likert ordinal scale: 1. ‘Not important at all’ to 5. ‘Very much Important’. |
|  | ADVC_PRFRD | The degree to which parents and friends were involved in deciding if to terminally leaving Israel or to repatriate to it | 2.2 | 0.9 | Ordinal scale of: 1.’Did not been involved’ to 5. ‘They were involved to a very great extent’. |
|  | ASST_PRS | The degree that parents (and his or her spouse's parents) routinely assist their offspring (e.g. in funding, child rearing and education) | 2.6 | 0.1 | Ordinal scale of: 1.’Do not assist’ to 5. ‘They are assisting to a very great extent’. |
|  | ASST_INST | Was the institution in which the scholar works at (or was employed right after his or her graduation/postdoc) assisting in searching for a job for his or her spouse? | 0.2 | 0.0 | Dichotomic nominal scale of: 0. 'No'. 1. 'Yes'. |
