# Supplementary material for: Return on capital? Determinants of counter-migration among early career Israeli STEM researchers
Source: PLoS One. 2019 Aug 8;14(8):e0220609. doi: 10.1371/journal.pone.0220609 (PMC6687145; doi:10.1371/journal.pone.0220609)
Supplement: S2 Appendix — (DOCX) [file pone.0220609.s002.docx]

**Caption:**

S2 Appendix: Bivariate correlation matrix between explanatory variables

**S2 Appendix**: Bivariate correlation matrix between explanatory variables

|  | A | B | C | D | E | F | G | H | I | J | K | L |
| --- | --- | --- | --- | --- | --- | --- | --- | --- | --- | --- | --- | --- |
| A | 1.000 | .242^**^ | -0.060 | 0.070 | 0.001 | -0.039 | 0.034 | 0.106 | 0.100 | .230^**^ | .375^**^ | -0.115 |
| B | .242^**^ | 1.000 | -0.067 | -0.045 | 0.048 | -0.041 | -0.003 | 0.030 | .137^*^ | 0.107 | .197^**^ | -.162^*^ |
| C | -0.060 | -0.067 | 1.000 | -0.054 | -0.111 | 0.050 | -0.112 | -0.051 | .277^**^ | -0.063 | -0.071 | 0.052 |
| D | 0.070 | -0.045 | -0.054 | 1.000 | .239^**^ | 0.145 | 0.068 | 0.061 | -0.043 | .232^**^ | 0.084 | 0.002 |
| E | 0.001 | 0.048 | -0.111 | .239^**^ | 1.000 | 0.116 | .148^*^ | -0.060 | -0.076 | 0.097 | -0.010 | 0.052 |
| F | -0.039 | -0.041 | 0.050 | 0.145 | 0.116 | 1.000 | .216^**^ | 0.070 | 0.067 | 0.062 | 0.005 | 0.100 |
| G | 0.034 | -0.003 | -0.112 | 0.068 | .148^*^ | .216^**^ | 1.000 | -0.131 | 0.033 | 0.061 | 0.045 | 0.032 |
| H | 0.106 | 0.030 | -0.051 | 0.061 | -0.060 | 0.070 | -0.131 | 1.000 | .167^*^ | 0.132 | .282^**^ | -0.137 |
| I | 0.100 | .137^*^ | .277^**^ | -0.043 | -0.076 | 0.067 | 0.033 | .167^*^ | 1.000 | .186^*^ | 0.128 | -0.045 |
| J | .230^**^ | 0.107 | -0.063 | .232^**^ | 0.097 | 0.062 | 0.061 | 0.132 | .186^*^ | 1.000 | .299^**^ | -0.081 |
| K | .375^**^ | .197^**^ | -0.071 | 0.084 | -0.010 | 0.005 | 0.045 | .282^**^ | 0.128 | .299^**^ | 1.000 | -0.095 |
| L | -0.115 | -.162^*^ | 0.052 | 0.002 | 0.052 | 0.100 | 0.032 | -0.137 | -0.045 | -0.081 | -0.095 | 1.000 |

**** Significant at the 1% level; **Significant at the 5% level; *Significant at the 10% level*

| **Key** |  |  |
| --- | --- | --- |
| A - OWN_HOUS | F - PARTSPS_ACD | K - ASST_PRS |
| B - ECO_STUS2 | G - PARTSPS_PROF | L - ASST_INST |
| C - LKINFO | H - PRTS_PRX |  |
| D - PART_ACD | I - CONCT |  |
| E - PART_PROF | J - ADVC_PRFRD |  |
